# Correction: Sequential conformational rearrangements in flavivirus membrane fusion

**DOI:** 10.7554/eLife.08656

**Published:** 2015-05-18

**Authors:** Luke H Chao, Daryl E Klein, Aaron G Schmidt, Jennifer M Peña, Stephen C Harrison

Chao LH, Klein DE, Schmidt AG, Peña JM, Harrison SC. 2014. Sequential conformational rearrangements in flavivirus membrane fusion. *eLife* **3**:e04389. doi: 10.7554/eLife.04389. Published 5 December 2014

We recently noticed errors in the units and a reported value in Figure 6B that occurred during revisions. In Figure 6B, the units for rate constants should have read s^−1^ and *k*_*ret*_: 4 × 10^−7^ ms^−1^ should have read *k*_*ret*_: 1.5 × 10^−7^ s^−1^. The MATLAB script (Source code 1) has been updated to avoid any ambiguity. We apologize for any confusion that may have occurred.

The article has been corrected accordingly.

